# Unilateral Giant Seborrheic Keratosis of the Nipple–Areola Complex in a Young Woman: A Case Report

**DOI:** 10.7759/cureus.101593

**Published:** 2026-01-15

**Authors:** Cristina Lizbeth Puntos-Guizar, Dolores Maribel Arellano-Vivero, Lucia Achell-Nava, Guadalupe Maldonado-Colin

**Affiliations:** 1 Dermatology, Centro Médico Nacional 20 de Noviembre, Mexico City, MEX; 2 Dermatology, Centro Medico Nacional 20 de Noviembre, Mexico City, MEX

**Keywords:** benign and malignant breast lesions, breast symptoms in primary care, breast ultrasonography, eruptive seborrheic keratosis, giant seborrheic keratosis

## Abstract

Seborrheic keratosis is a common benign epidermal tumor that affects individuals of various ages and may arise on both sun-exposed and non-sun-exposed areas. Involvement of the nipple-areola complex is rare and may pose a diagnostic challenge due to its clinical resemblance to malignant or premalignant breast conditions, particularly Paget disease of the breast. We report the case of a 36-year-old woman who presented with a long-standing, progressively enlarging unilateral pigmented verrucous lesion involving the left nipple-areola complex, with onset and marked growth during two consecutive pregnancies. Clinical examination, dermoscopic evaluation, and breast imaging were performed, followed by histopathological analysis, which confirmed the diagnosis of acanthotic, pigmented seborrheic keratosis without evidence of malignancy. Given the benign nature of the lesion and patient preference, conservative management was selected with close dermatologic follow-up. This case highlights the importance of considering seborrheic keratosis in the differential diagnosis of nipple-areola lesions, even in young patients, and underscores the essential role of histopathological examination in establishing an accurate diagnosis and guiding individualized management.

## Introduction

Seborrheic keratosis represents a common benign proliferation of the epidermis that may occur across a wide age range, although it is more frequently observed in middle-aged and older adults [1]. These lesions typically arise on the trunk, face, or extremities, but may also occur in non-sun-exposed areas. Involvement of the nipple-areola complex is uncommon and may raise concern for malignant disease because of its atypical location and variable clinical appearance [2].

Lesions affecting the nipple-areola complex warrant particular attention because they may clinically resemble malignant or premalignant conditions, most notably Paget disease of the breast, which classically presents as an eczematous, erythematous, or ulcerated lesion and is often associated with underlying breast carcinoma [3,4]. Other important differential diagnoses include melanoma and basal cell carcinoma. For this reason, a biopsy is frequently required in this anatomical location to establish a definitive diagnosis. Accurate diagnosis relies on careful clinicopathological correlation, supported by dermoscopy, breast imaging, and histopathological examination [1,3].

## Case presentation

A 36-year-old woman from Hidalgo, Mexico, with no significant past medical history, was referred for evaluation of a unilateral lesion involving the left nipple-areola complex. She denied smoking, alcohol consumption, or illicit drug use. There was no personal or family history of malignancy. Surgical history included two cesarean sections (2017 and 2019) and a tubal ligation.

**Figure 1:**
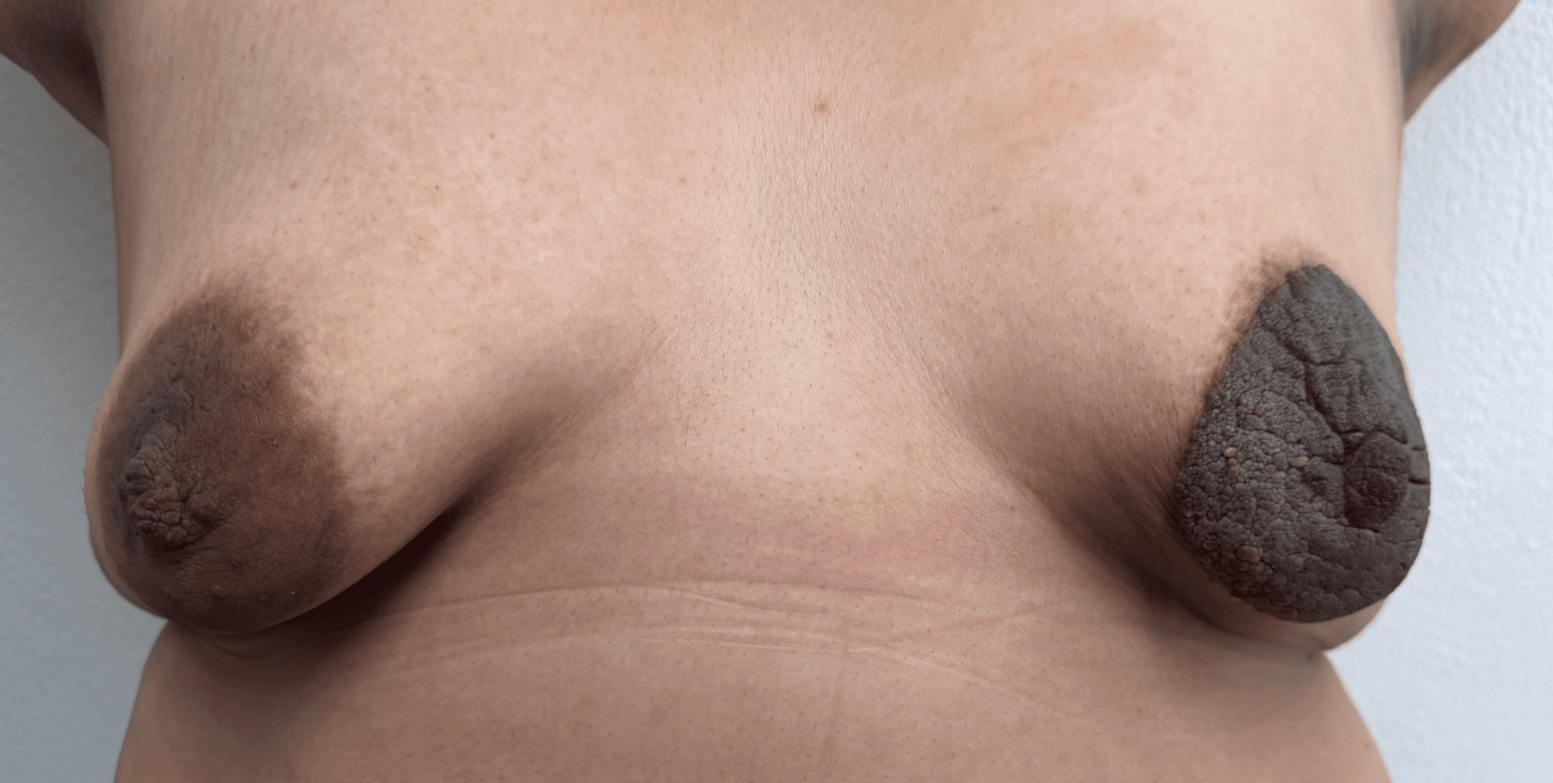
Clinical overview of a unilateral hyperpigmented, verrucous lesion involving the left nipple–areola complex, measuring approximately 10 × 10 cm.

The lesion first appeared in February 2017 during the first trimester of her first pregnancy as a small pigmented plaque on the areola and progressively enlarged over time. During her second pregnancy in 2019, the lesion increased notably in size and thickness, eventually involving most of the nipple (Figure 1). The lesion persisted chronically and was incidentally identified during a routine health screening in 2024, prompting referral to specialized care.

The patient denied associated symptoms such as pruritus, pain, nipple discharge, ulceration, bleeding, erosion, or nipple retraction.

Initial breast imaging revealed no suspicious masses or axillary lymphadenopathy. An incisional biopsy was performed in March 2025, targeting the thickest and clinically representative area of the lesion to minimize sampling error given its large size. Histopathological analysis demonstrated acanthotic, pigmented seborrheic keratosis. The patient was subsequently referred to a tertiary care center for further evaluation.

**Figure 2:**
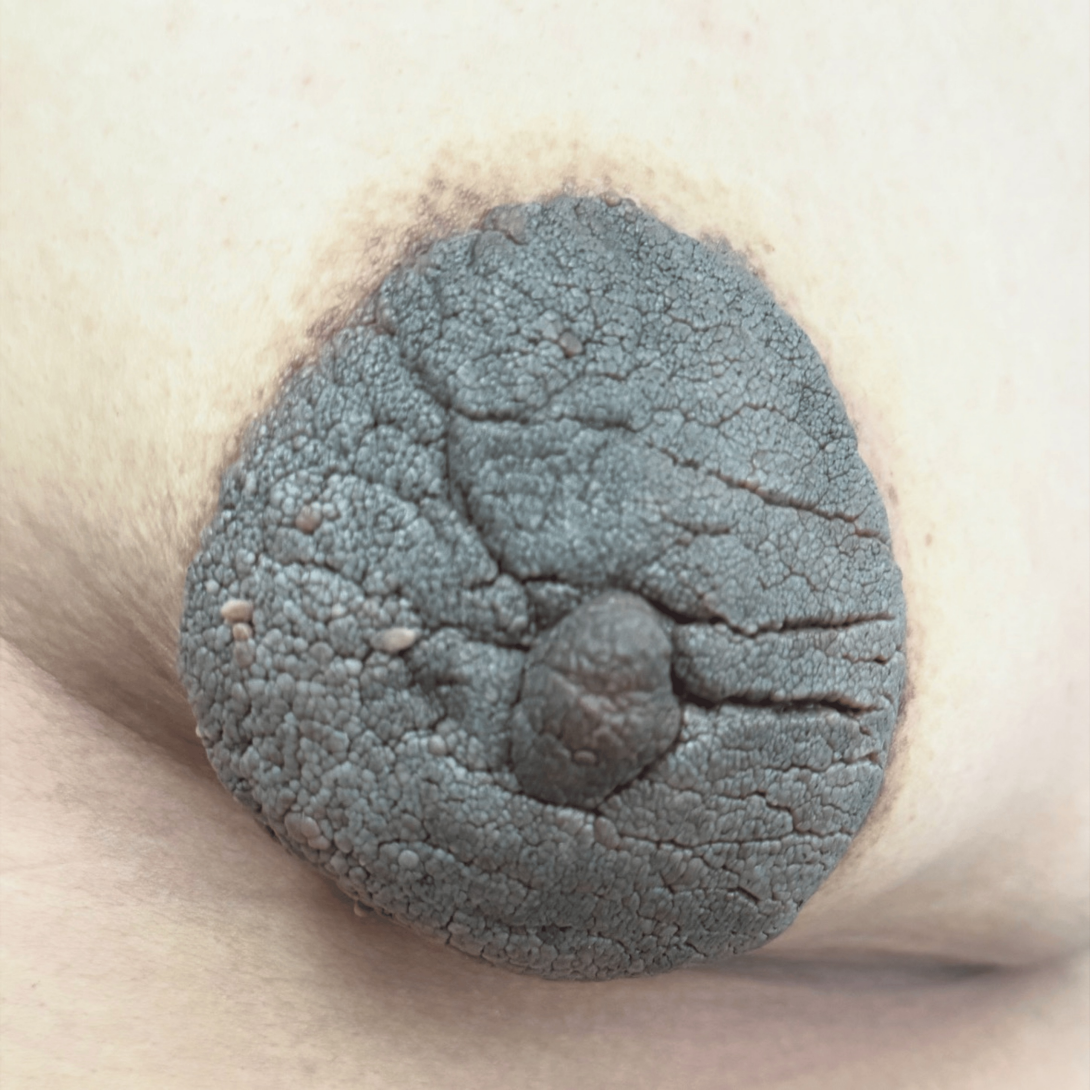
Close-up view highlighting the verrucous surface and well-defined borders of the lesion.

On physical examination, the patient was asymptomatic with an Eastern Cooperative Oncology Group (ECOG) performance status of 1. Breast examination showed a hyperpigmented, thickened, verrucous unilateral lesion involving the left nipple-areola complex, measuring approximately 10 × 10 cm, with well-defined borders and no palpable underlying mass (Figure 2). The right breast and both axillae were unremarkable.

**Figure 3:**
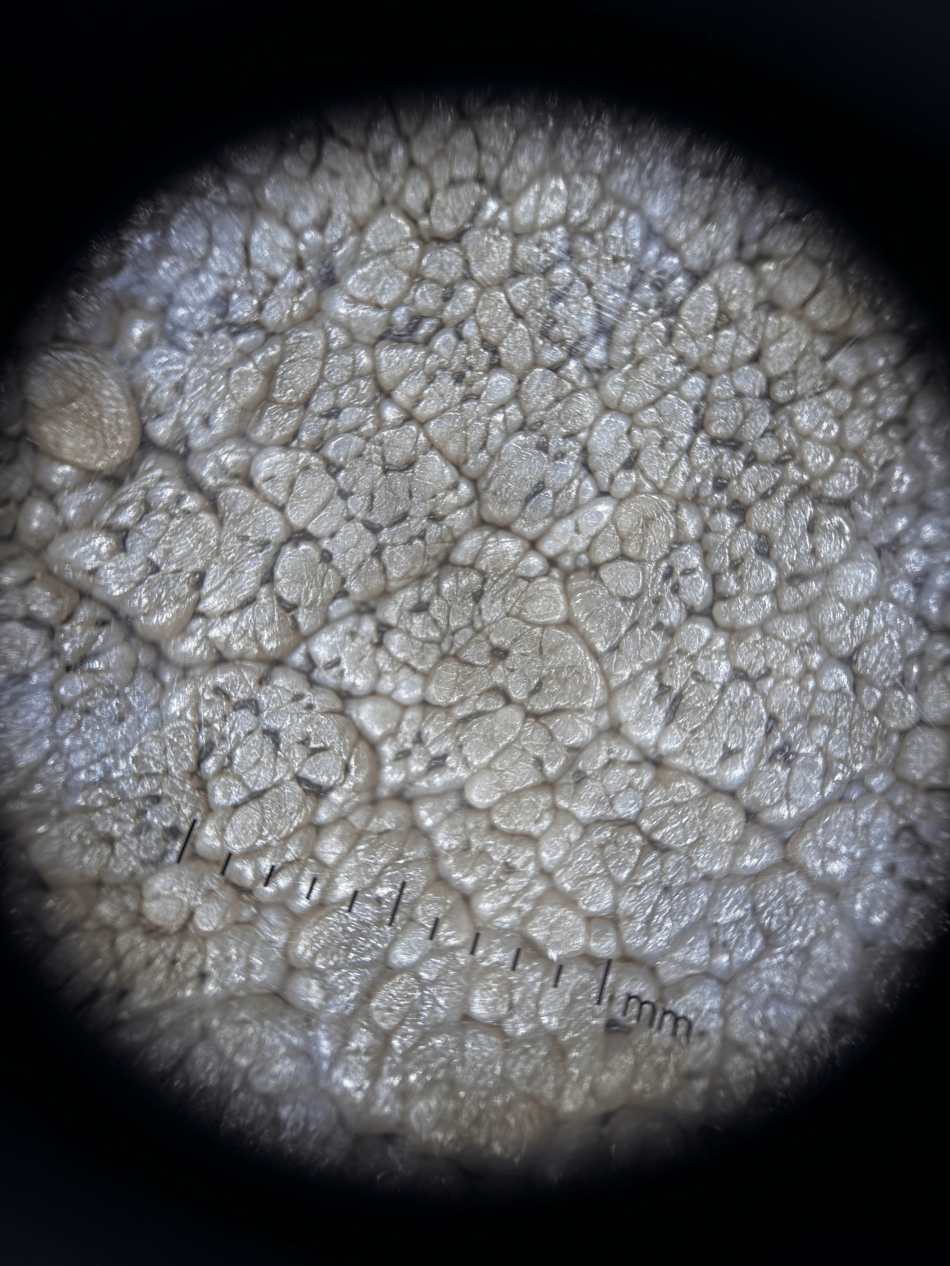
Cerebral pattern, follicular openings, and milium cysts are visible.

**Figure 4:**
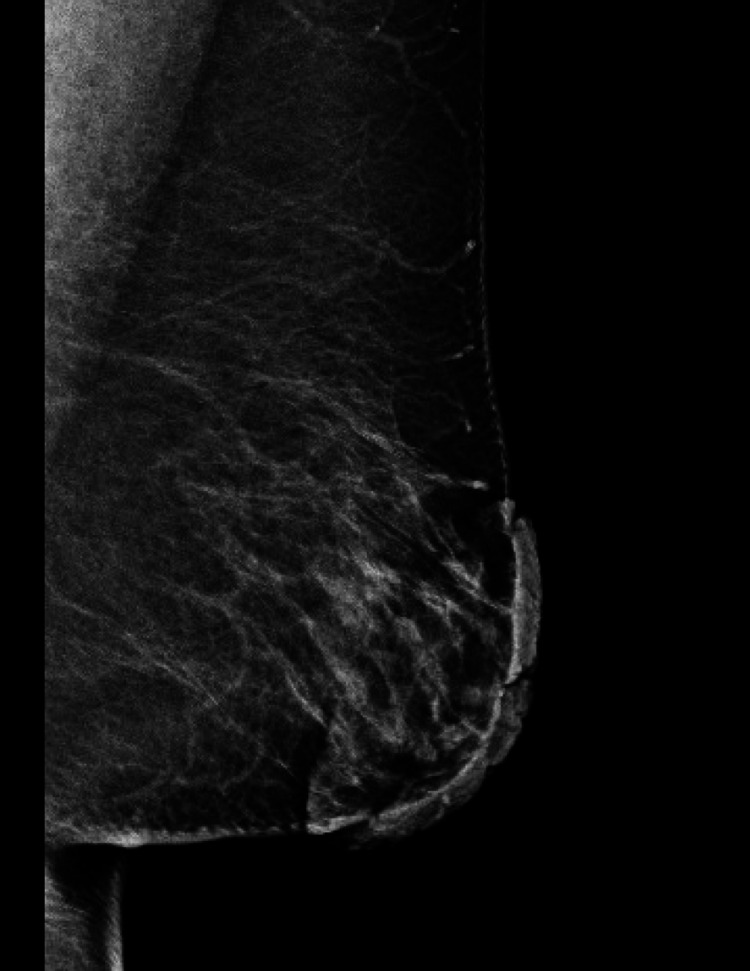
The breast tissue has a heterogeneous echo structure. Scattered areas of fibroglandular density are observed. Thickening of the skin of the left nipple-areola complex.

**Figure 5:**
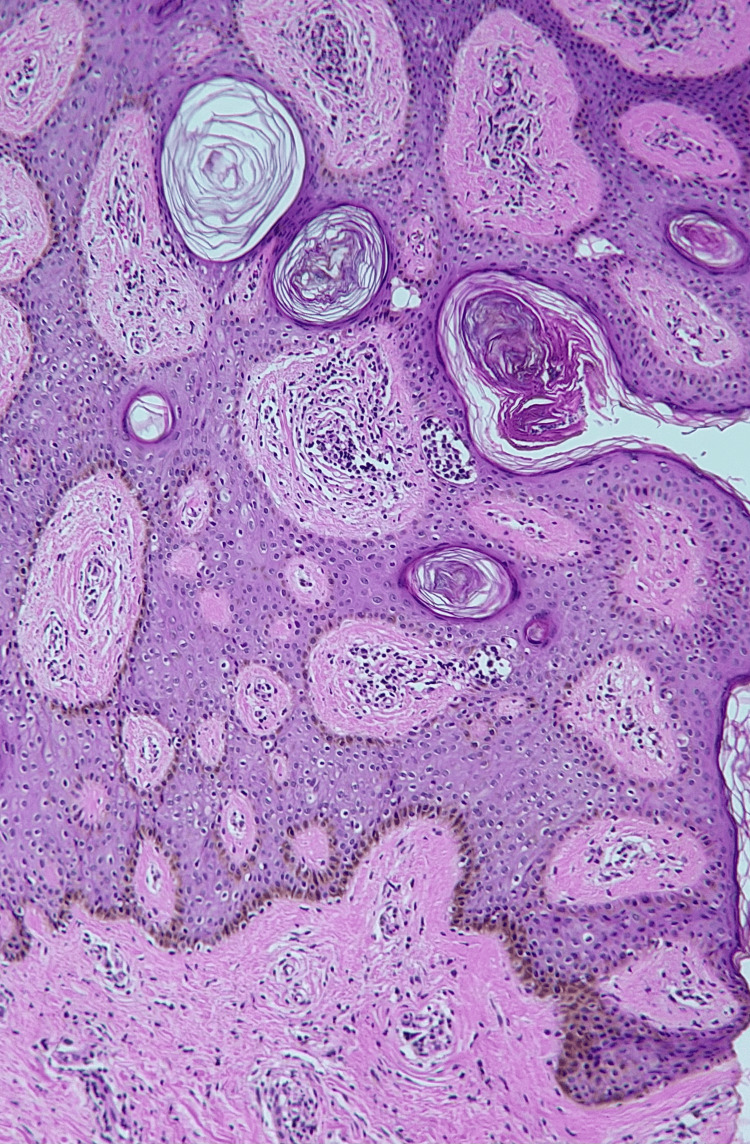
Acanthoid and pigmented seborrheic keratosis.

Dermoscopy revealed a cerebriform pattern with comedo-like openings and milia-like cysts (Figure 3). Mammography and breast ultrasound were classified as BI-RADS 2, demonstrating only cutaneous thickening of the left nipple-areola complex without underlying parenchymal lesions or axillary adenopathy (Figure 4). Histopathological slide review at the tertiary center confirmed seborrheic keratosis without evidence of dysplasia or malignancy (Figure 5).

Management and outcome

Given the confirmed benign histopathological diagnosis, the absence of imaging findings suggestive of malignancy, and the patient’s preference, conservative management was selected. Treatment consisted of general skin care measures, photoprotection, and topical urea 20%. The patient reported mild irritation related to friction and hygiene difficulties due to the lesion size, with partial symptomatic improvement during follow-up.

Alternative therapeutic options, including surgical excision or ablative modalities such as laser therapy, were discussed as potential future interventions should symptoms persist, cosmetic concerns increase, or diagnostic uncertainty arise. Owing to the absence of malignant features, no immediate surgical intervention was indicated, and the patient was discharged from tertiary-level care with planned dermatologic follow-up.

## Discussion

Seborrheic keratosis is among the most prevalent benign epidermal tumors; however, involvement of the nipple-areola complex remains distinctly uncommon [1,2]. Although these lesions are more frequently reported in middle-aged or elderly individuals, seborrheic keratoses have also been described in younger patients, including familial cases, which should be recognized to avoid diagnostic bias.

The principal differential diagnosis is Paget disease of the breast, an uncommon manifestation of underlying breast carcinoma that typically presents as an eczematous, erythematous, or ulcerated nipple lesion. This condition is often accompanied by symptoms such as pruritus, burning sensation, nipple discharge, or retraction [4]. In contrast, seborrheic keratosis classically appears as a well-circumscribed, verrucous, or plaque-like lesion with a characteristic “stuck-on” appearance. Nevertheless, pigmentation, surface irregularity, or secondary inflammatory changes may obscure these classic features, particularly when lesions arise in atypical locations such as the nipple-areola complex [1].

Additional differential diagnoses that should be considered include melanoma, basal cell carcinoma, Bowen disease, and verrucous carcinoma [2]. Dermoscopy serves as a useful noninvasive adjunct by demonstrating features suggestive of seborrheic keratosis, including milia-like cysts, comedo-like openings, and a cerebriform or fissured pattern [3]. Despite its utility, dermoscopy alone is insufficient to reliably exclude malignancy, as overlapping features may be present. Consequently, histopathological examination remains the diagnostic gold standard, especially for large, symptomatic, or anatomically atypical lesions [1,3].

Hormonal factors have been proposed to influence the development or progression of seborrheic keratoses. Pregnancy-related hormonal changes may promote epidermal proliferation through estrogen- and progesterone-mediated pathways, as well as increased expression of growth factors and signaling pathways such as FGFR3 and PI3K/AKT [5]. In the present case, the onset and marked progression of the lesion during two consecutive pregnancies support a potential hormonal contribution to lesion growth.

From a therapeutic standpoint, seborrheic keratosis generally does not require treatment unless lesions are symptomatic, cosmetically distressing, or associated with diagnostic uncertainty [1]. However, when the nipple-areola complex is involved, a low threshold for histopathological confirmation is warranted due to the significant clinical and psychological implications of overlooking a malignant diagnosis [2,4]. While treatment options such as surgical excision, cryotherapy, curettage, or laser ablation may be considered in selected cases, conservative management is appropriate once malignancy has been confidently excluded, as demonstrated in this patient.

This case highlights the importance of a multidisciplinary approach involving dermatology, surgical oncology, and radiology. Such collaboration facilitates accurate diagnosis, minimizes unnecessary aggressive interventions, and provides reassurance to patients presenting with lesions in anatomically and emotionally sensitive areas.

## Conclusions

Seborrheic keratosis should be included in the differential diagnosis of lesions involving the nipple-areola complex, even in young patients and in the absence of traditional risk factors for breast malignancy. Lesions in this anatomically sensitive region may closely mimic malignant or premalignant conditions, particularly Paget disease of the breast, leading to diagnostic uncertainty.

This case underscores the essential role of histopathological examination in establishing a definitive diagnosis and excluding underlying malignancy. Although conservative management was appropriate in this patient, treatment decisions should be individualized, and surgical or ablative interventions may be considered for extensive or symptomatic lesions depending on clinical evolution and patient preference.
